# Diversity and Dynamics of Epidermal Microbes During Grape Development of Cabernet Sauvignon (*Vitis vinifera* L.) in the Ecological Viticulture Model in Wuhai, China

**DOI:** 10.3389/fmicb.2022.935647

**Published:** 2022-06-30

**Authors:** Ru-teng Wei, Ning Chen, Yin-ting Ding, Lin Wang, Fei-fei Gao, Liang Zhang, Yi-hui Liu, Hua Li, Hua Wang

**Affiliations:** ^1^College of Enology, Northwest A&F University, Xianyang, China; ^2^Shaanxi Engineering Research Center for Viti-Viniculture, Xianyang, China; ^3^China Wine Industry Technology Institute, Zhongguancun Innovation Center, Yinchuan, China

**Keywords:** high throughput sequencing, ecological viticulture, grape, microbial diversity, developmental stage, vineyard weather

## Abstract

Grapevine-related microorganisms affect the health and yield of grapes, the metabolic pathways of the fermentation process, and the regional characteristics of wine. However, the diversity of epidermal microorganisms during the development of berries under the ecological viticulture model has not been described in detail. In this study, high-throughput amplicon sequencing technology was used to perform ITS and 16S sequencing of Cabernet Sauvignon epidermal microbes at different developmental stages in the Wuhai region to investigate the succession of epidermal microbes and their response to developmental stages and vineyard weather. The results showed that the diversity of fungi and bacteria decreased during development. Epidermal microorganisms recruited members according to their developmental stages, but retained the core taxa, such as the fungi genera *Alternaria*, *Jattaea*, and *Jattaea* and the bacteria genera *Brevundimonas*, *Sphingomonas*, *Acinetobacter*, and *Pseudomonas*. In addition, the microbial diversity was associated with specific meteorological parameters, implying that there was a connection between the environmental conditions of the vineyard and the microbial distribution pattern such as the fungus genus *Filobasidium* was positively correlated with relative humidity and negatively correlated with average high temperature, average low temperature, and average ground temperature; the bacterium genus *Lactobacillus* was positively correlated with sunlight time, and negatively correlated with relative humidity. In conclusion, this study can help vineyard managers understand the microbial consortia associated with particular diseases, and also the dynamics of infection processes in order to take preventive actions, especially at the most critical moments.

## Introduction

Wine is unique because it is essentially a natural product, which is a result of ordered and complex biochemical transformations including the ripening of grape and the metabolism of microbes from fermentation to aging (Liu et al., 2017; Carpena et al., 2020; Echave et al., 2021). In winemaking, “microbial terroir” refers to the contribution of microorganisms in a region to the characteristics of wine, and is a process that starts in the vineyards and then develops along the different stages of fermentation (Bokulich et al., 2014; Comitini et al., 2017; Wei et al., 2022a). Thus, the microbiological aspects of wine production are influenced by the vineyard ecosystem and not simply by the winery and fermentative processes (Grangeteau et al., 2017; Liu et al., 2019).

Grapevine hosts a variety of microorganisms (fungi, yeast, and bacteria) on and inside organs and their surrounding soil (Wei et al., 2022b). Among these, inhabitants are both harmful and beneficial microbes that are involved in crucial functions such as plant nutrition and plant resistance to biotic and abiotic stresses, hence in plant growth promotion, fruit yield, disease resistance, and survival (Barata et al., 2012; Wei et al., 2022d). Most studies have focused on a series of plant pathogenic fungi that affect grapes, including *Erysiphe necator*, *Botrytis cinerea*, and *Plamospara viticola* – the causative agents of grapevine powdery mildew, gray rot, and downy mildew, respectively. In addition, grapes may bear saprophytic molds such as *Aspergillus* spp., *Cladosporium* spp., and *Penicillium* spp., which are directly responsible for several grape rots and are indirectly involved in food spoilage because they produce mycotoxins (Martins et al., 2014). These fungi are unable to grow in wines, and their effect on wine quality is due to grape damage (Barata et al., 2012). In addition, some of these microorganisms are even considered as natural biocontrol agents due to their ability to protect the plant against phytopathogens and enhance the natural plant defenses (Pinto et al., 2014). *Trichoderma* is one of the most studied and applied fungal biocontrol agents. The benefits of these microorganisms to the plant include suppression of pathogens, growth promotion, enhanced nutrient availability, and induction of resistance. The biological activity is related to the variety of metabolites that they produce. These metabolites have been found to directly inhibit the pathogens, increase the disease resistance, and enhance the plant growth (Pascale et al., 2017).

In recent studies, a large number of literature have reported microbial diversity associated with vineyards. In addition, high-throughput sequencing technologies enable the detection and quantification of microorganisms present in vineyard soil and grapes, as well as its transformation later in winery (Bokulich et al., 2016; Morgan et al., 2017; Liang et al., 2019). High-throughput analysis of the grapevine phyllosphere, flowers and grape berry surfaces, demonstrated that the bacterial communities were predominated by *Proteobacteria* followed by *Firmicutes*, *Actinobacteria*, *Acidobacteria*, and *Bacteroidetes* (Perazzolli et al., 2014; Pinto et al., 2014, 2015; Portillo et al., 2016). The relative abundances of the groups vary depending on the plant tissue or organ. Dominant taxa include members of the genera *Pseudomonas*, *Sphingomonas*, *Frigoribacterium*, *Curtobacterium*, *Bacillus*, *Enterobacter*, *Acinetobacter*, *Erwinia*, *Citrobacter*, *Pantoea*, and *Methylobacterium* (Bokulich et al., 2014; Perazzolli et al., 2014; Pinto et al., 2015; Zarraonaindia et al., 2015; Portillo et al., 2016). The fungal diversity is very similar at the phylum level, mainly composed of *Ascomycetes* and *Basidiomycetes*. Other phyla such as *Zygomycota* and *Chytridiomycota* are only present in low abundance. Frequently encountered genera of filamentous fungi include *Aspergillus*, *Alternaria*, *Penicillium*, *Cladosporium*, *Lewia*, *Davidiella*, *Erysiphe*, and *Botrytis* and the yeast-like fungus, *Aureobasidium pullulans*, while the yeast genera include *Hanseniaspora*, *Issatchenkia*, *Pichia*, *Candida*, *Rhodotorula*, *Lachancea*, *Metschnikowia*, *Cryptococcus*, *Filobasidiella*, *Sporobolomyces*, and *Torulaspora* (Bokulich et al., 2014; Pinto et al., 2014; Wang et al., 2015; Filippis et al., 2017; Liu and Howell, 2021). The grape berry surface is, nevertheless, a natural habitat of microorganisms. Diversity and stability of the grape epidermal microorganisms are strongly associated with numerous factors, such as vineyard geography (altitude, latitude, and longitude; Gao et al., 2019), climatic conditions (rain, temperature, humidity, and maturity period; Liu and Howell, 2021; Wei et al., 2022c), grape variety, and viticultural practice (herbicides, fertilizers, pesticides, and fungicides used; Pinto et al., 2014).

The new ecological approach to viticulture with emphasis on ecologically sound grape production views grapevines as part of a complex agroecosystem where many organisms co-exist and interact (Likar et al., 2017). In particular, this approach recognizes the importance of interactions between the microbial communities and the plants, as these influence the growth, physiology, and yield of the grapevines (Compant et al., 2019). Although the new ecological approach to viticulture recognizes the importance of grapevine interactions with microbial communities, there remains less knowledge available on diversity and dynamics of grape microbial communities under ecological viticulture models. Therefore, we collected Cabernet Sauvignon berries at different developmental stages from ecological vineyards located in the Wuhai region, characterized the fungal and bacterial diversity of grape epidermis by amplicon sequencing, and further investigated the succession of epidermal microorganisms and the co-occurrence model of meteorological parameters and epidermal microorganisms.

## Materials and Methods

### Location Description and Sampling

In this study, from June to October in 2020, berry samples were collected from the Sunshine Tianyu International Winery Cabernet Sauvignon vineyard located in the Wuhai region (Inner Mongolia, China; Supplementary Figure 1). The vineyard adopts an ecological viticulture management model. No chemical fertilizers or insecticides are applied for pest or disease control during the grape growing season, and weeds are controlled *via* manually weeding every month. The cultivation frame adopts Modified VSP (All the vines were trained to a slope trunk with a vertical shoot positioning trellis system), which is convenient for the main vines to be buried in the soil in winter (Supplementary Figure 1). The sunshine hours (h), precipitation (mm), average low temperature (°C), average high temperature (°C), average ground temperature (°C), and relative humidity (%) were measured weekly. Meteorological data originated from the vineyard’s meteorological equipment.

To evaluate the changes in the microbial community during grape development, berry samples from the following five stages were collected aseptically: fruit setting (A), early veraison (B), end veraison (C), mid maturity (D), and harvest (E), corresponding to stages 31, 35, 36, 37, and 38 in the improved E-L system. In the Cabernet Sauvignon vineyard, a relatively flat plot with an area of approximately 1.5 ha and a regular topography was selected for sampling. A total of 3 biological replicates were sampled at each developmental berry stage, and each replicate was collected from 5 sample points fixed in the sampling plot that could cover the area of the sampling plot. Considering the heterogeneity of the tested grapes, the berries were collected from the upper, central, and lower part of the cluster at each sample point, both from the sun-exposed and shaded side. A total of 15 healthy grape berry samples were collected. All berry samples were immediately stored in sterile bags, transported to the laboratory on dry ice, and stored at −80°C before molecular analysis.

### DNA Extraction and Sequencing

The sample was thawed at 28°C for 30 min. An amount of 10 g of the sample was aseptically transferred to a polypropylene test tube containing 100 ml of washing solution (0.1 M potassium phosphate buffer, pH 7.0), and ultrasonic treatment was performed in an ultrasonic cleaning tank at a frequency of 40 kHz for 10 min. The sediment filtered was used to extract the microbial genome DNA. The process was performed using the methods described by Gao et al. (2019). Genomic DNA was submitted to Gene Denovo company (Guangzhou, China) for amplification. The primers ITS3_KYO2F (GATGAAGAACGYAGYRAA) and ITS4-2409R (TCCTCCGCTTATTGATATGC) were used to amplify the fungal ITS2 region, and the primers 341F (CCTACGGGNGGCWGCAG) and 806R (GGACTACHVGGGTATCTAAT) were used to amplify the V3–V4 region of the bacterial 16S rDNA gene. The PCR process was performed under the following conditions: 94°C for 5 min, 94°C for 30 s, 55°C for 30 s, and 28 cycles of 72°C for 1 min, and the final extension was performed at 72°C for 10 min (Zhang et al., 2017). The amplicon was recovered from 2% agarose gel and purified using the AxyPrep DNA Gel Extraction Kit (Axygen Biosciences, Union City, CA, United States) according to the manufacturer’s instructions and quantified using an ABI StepOnePlus Real-Time PCR system (Life Technologies, Foster City, United States; Zhou et al., 2019). The purified amplicons were subjected to paired-end sequencing (HiSeq 2500, PE250) on the Illumina platform according to standard operations.

We used FASTP (version 0.18.0) and FLSAH (version 1.2.11) to perform quality filtering and sequence merging on the raw fastq files of 15 grape samples to obtain Tags. Then, according to the QIIME (version 1.9.1) Tags Quality Control process, low-quality tags were filtered to obtain high-quality Clean Tags. Based on the reference database (Quast et al., 2013), the UCHIME Algorithm was used to detect and delete the chimeras of tags to obtain effective Clean Tags (Edgar et al., 2011). Finally, Clean Tags were assigned to the same OTUs (Operational Taxonomic Units) according to the similarity of ≥97% by using UPARSE (version 9.2.64; Edgar, 2013). The tag sequence with the highest abundance was selected as the representative sequence of each OTU. For each representative sequence, the classification information was annotated using the SIlVA database based on the mothur algorithm (Quast et al., 2013). The sequence data are available in NCBI under BioProject number PRJNA766154.

### Data Analysis

According to the alpha index, one-way analysis of variance (ANOVA) and the Duncan test (*p* < 0.05) were used to analyze whether the sample diversity at different stages contained statistically significant differences. The statistical analyses were performed using the SPSS 25.0 (IBM, United States) software. Principal component analysis (PCoA) was used to evaluate the distribution pattern of grape epidermis microorganisms in different stages based on beta-diversity calculated by the Bray-Curtis distance with the “labdsv” package. The relatively abundant species (average relative abundance > 0.01%) were selected for network analysis, and those statistically significant and robust (Spearman correlation coefficient, *r* ≥ 0.6; *p* < 0.05) correlations were considered effective co-occurrence by Spearman correlation coefficient and were visualized by Cytoscape. Linear discriminant analysis effect size (LEfSe) was used to investigate the significant taxonomic differences of fungi and bacteria at different developmental stages. The OTU table was filtered to contain only the OTU with the relative abundance greater than 0.1% to reduce the LEfSe complexity. The factorial Kruskal–Wallis sum-rank test was used to identify taxa with significant differential abundances between classes, followed by the logarithmic LDA score (threshold = 4.0) to estimate the effect size of each discriminative feature. The correlation between the environmental characteristics and microbial community composition was examined by canonical correspondence analysis (CCA). The co-occurrence/interaction patterns between grape epidermis microorganisms and weather conditions during the growing season were explored using network analysis with Cytoscape. Correlation matrices were calculated with all possible pair-wise Spearman’s rank correlations between selected taxa and weather indexes. Correlations with a Spearman correlation coefficient *r* ≥ 0.5 and *p* < 0.05 were considered statistically robust. After the basic analysis, the visualization diagram was finally drawn using the package in RStudio (version 2.15.3).

The core microbiome was determined by abundance-occupancy distribution, including highly abundant and ubiquitous taxa. For the core microbiome of grape epidermis, the core OTU was the top 10% abundance of all samples which accounts for 50% of all samples or 100% at any developmental stage (average relative abundance ≥ 0.01%). Dynamics and successions of the core microbiome were illustrated by alluvial diagrams using the “ggalluvial” package in “ggplot2”.

## Results

### Evaluation of the Diversity and Richness of Microbial Populations in Grape Epidermis

**FIGURE 1:**
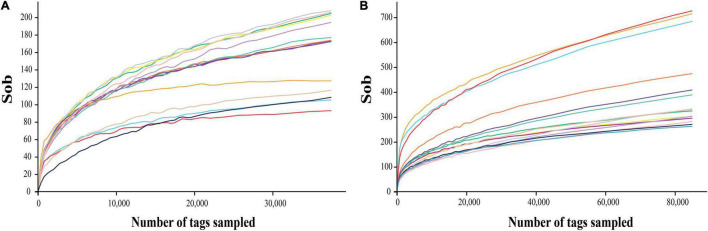
Rarefaction curves of fungi **(A)** and bacteria **(B)** in each sample with 97% similarity.

The deep sequencing of microbial communities was originated from a total of 1,286,519 sequences, of which 1,283,205 sequences passed the Quality Control filters, which represented 99.7% of the obtained sequences (Table 1). For fungal microorganisms, a total of 648,234 sequences were obtained, while for bacterial microorganisms, a total of 634,971 sequences were obtained (Table 1). The number of reads per sample ranged from 122,648 to 132,602 sequences. All the high-quality sequence reads were grouped with 97% homology, and generated a total of 1,113 OTUs for fungi, and 2,175 for bacteria. On average, fungi and bacterial microorganisms generated 223 ± 17 and 435 ± 12 OTUs, respectively. The diversity of fungal and bacterial communities was compared between samples by rarefaction curve analysis (Figure 1). The rarefaction curve tended to be flat, indicating that deep sequencing provided good overall OTU coverage. For each sample, the expected richness (Chao1 index) has been determined. In the current analysis, we have predicted a total richness ranging from 223 ± 17 (fungal community) to 435 ± 12 (bacterial community). By comparing the obtained number of OTUs with its predicted Chao1, we were able to determine the coverage of our experiments. The richness estimators indicated that 80.6 ± 3.2 and 62.9 ± 1.5% of the fungal and bacterial community diversity were uncovered, respectively (Table 1). Therefore, we realized that despite revealing the complex and rich microbial structure, there still exists a hidden biodiversity in grapes that cannot be exposed.

**TABLE 1:** Total sequences obtained for fungi and bacterial communities for all samples.

Time points	Target region	Raw reads	Clean reads	OTU that passed the blast	CHAO 1	ACE	Coverage (%)^a^
A	ITS2	132,689 ± 3666	132,602 ± 3178	148 ± 22.48	141 ± 24.31	135 ± 20.63	100
	V3-V4	122,896 ± 3791	122,648 ± 3700	774 ± 28.57	1145 ± 51.41	1228 ± 53.41	67.6
B	ITS2	132,164 ± 3433	132,014 ± 3260	279 ± 36.38	375 ± 51.76	377 ± 47.58	74.4
	V3-V4	127,123 ± 6108	126,752 ± 6130	327 ± 11.06	535 ± 53.31	494 ± 48.05	61.1
C	ITS2	125,810 ± 4372	125,682 ± 4213	243 ± 25.11	317 ± 64.49	316 ± 60.71	76.7
	V3-V4	131,382 ± 7144	130,684 ± 6966	440 ± 48.99	741 ± 78.46	775 ± 45.71	59.4
D	ITS2	127,882 ± 7039	127,725 ± 6972	302 ± 12.85	421 ± 54.24	421 ± 15.49	71.7
	V3-V4	124,082 ± 2662	123,527 ± 2593	332 ± 33.85	614 ± 92.43	682 ± 13.73	54.1
E	ITS2	130,586 ± 7710	130,211 ± 7660	141 ± 16.46	132 ± 15.96	133 ± 13.09	100
	V3-V4	131,905 ± 3131	131,360 ± 3057	302 ± 38.21	424 ± 50.47	443 ± 56.67	71.2
Σ	ITS2	649,131 ± 12081	648,234 ± 11829	1113 ± 88.06	1386 ± 156.51	1382 ± 149.96	80.6 ± 3.2
	V3-V4	637,388 ± 12698	634,971 ± 12345	2175 ± 60.53	3459 ± 163.51	3622 ± 195.02	62.9 ± 1.5
TOTAL		1,286,519	1,283,205	3,288	4,845	5,004	

*Values are presented as mean ± standard error (n = 3).*

*^a^The coverage obtained is determined as the ratio between the observed OTUs and the estimated Chao 1 (OTUs/Chao 1).*

*A–E represent stages of grape berry development. A, fruit setting; B, early veraison; C, end veraison; D, mid maturity; E, harvest.*

### Diversity Changes During Grape Development

**FIGURE 2:**
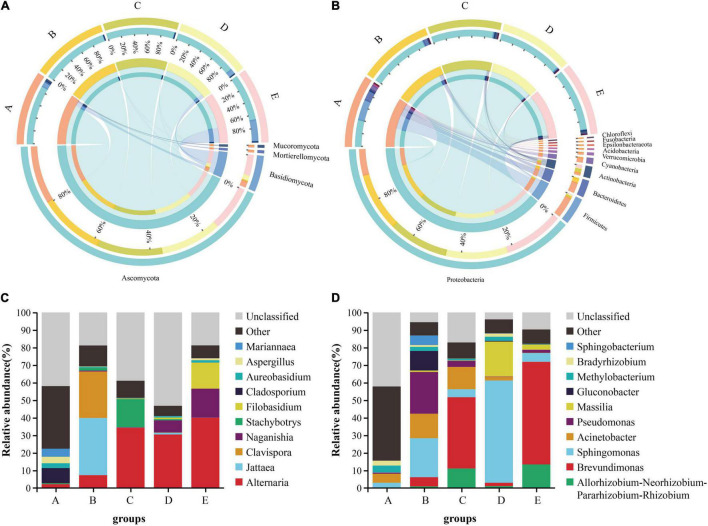
The differences in the microbial communities of Cabernet Sauvignon grapes at different growth stages. The difference between fungi **(A)** and bacteria **(B)** communities at the phylum level (relative abundance > 0.01%). The relative abundance of fungi **(C)** and bacteria **(D)** communities at the genus level (Shown top10). A, fruit setting; B, early veraison; C, end veraison; D, mid maturity; E, harvest.

Grape microbial community compositions evolved over time. At the phylum level for fungal, most significant was the massive colonization of the grapes by Ascomycota, and it existed in high abundance at each stage, especially the veraison (B stage: 99.19%, C stage: 99.26%); the second highest abundance was shown by Basidiomycota, especially at the harvest (31.48%), but the veraison was the lowest (Figure 2A). At the genus level, the dominant taxa *Alternaria* was found to be massively enriched in the grape epidermis, which increased as the development stage proceeded (Figure 2C). At the phylum level for bacterial, most significant was the massive colonization of the grapes by Proteobacteria, and it existed in high abundance at each stage; the second highest abundance was shown by Firmicutes, mainly enriched at the fruit setting (30.89%; Figure 2B). At the genus level, the dominant taxa *Brevundimonas* was found to be massive enriched in the grape epidermis, which increased as the development stage proceeded, but decreased at the harvest (Figure 2D).

**FIGURE 3:**
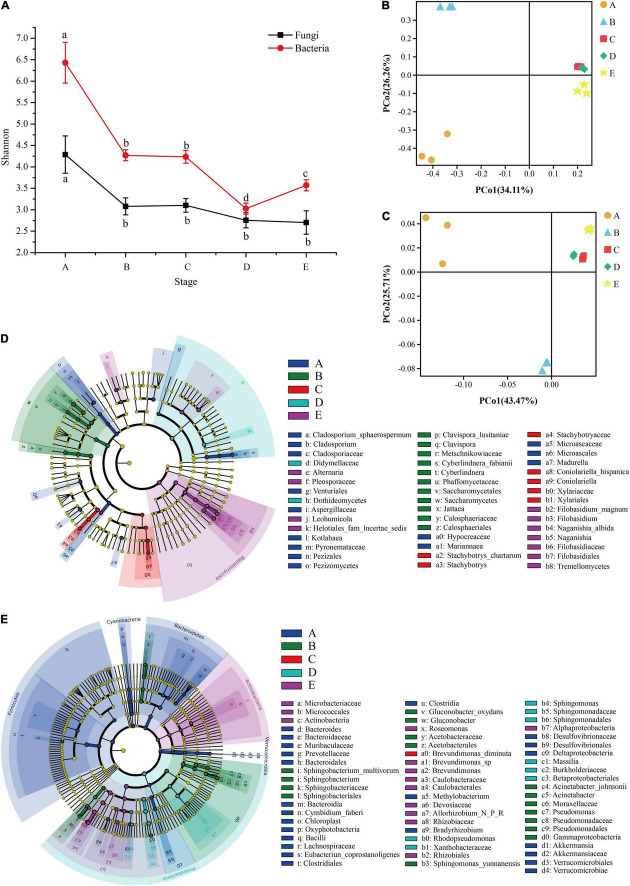
The microbial diversity of the epidermis changes during grape development. The line graph **(A)** shows the changing trend of fungal and bacterial α-diversity (Shannon index) during grape growth. PCoA plots based on the fungal Bray-Curtis distance **(B)** and the bacterial weighted UniFrac distance **(C)** of samples at different stages. LEfSe analysis revealed fungal **(D)** and bacterial **(E)** taxa with significant differences at different stages. A, fruit setting; B, early veraison; C, end veraison; D, mid maturity; E, harvest.

Microbial diversity and similarity of grape epidermis changed with the growth stage. According to the measurement of the Shannon index, the diversity of fungal and bacterial communities in this study tended to decline over time. The Shannon index integrates the richness and evenness of the community. The fungal community declined significantly from the A stage to the B stage, and then the decline was not significant (Figure 3A). The bacterial community also decreased significantly from the A stage to the B stage, and did not change significantly during the veraison process. When it came to maturity, it first declined and then increased (Figure 3A). Interestingly, the diversity of fungal and bacterial communities changed significantly at the B stage.

In addition, it was found that there were significant differences in the composition of fungal and bacterial communities over time (Supplementary Table 1, β-diversity, PERMANOVA; Bray-Curtis, *R*^2^ = 0.7036, and *P* = 0.001; Bacterial weighted UniFrac, *R*^2^ = 0.8588, and *P* = 0.001). PCoA showed that the samples at different stages were clearly separated, and the first two principal axes (PC) of the fungal and bacterial communities explained 60.37 and 69.18% of the total variation, respectively (Figures 3B,C). LEfSe analysis further confirmed that the pattern was related to the significant association between the species taxa and sample (Kruskal–Wallis rank-test, α < 0.05). LEfSe showed that there were 46 fungi taxa with significant differences. Among them, there were 14 taxa for the A stage (e.g., *Cladosporium*, *Aspergillaceae*, and *Microascales*), 11 for the B stage (e.g., *Clavispora*, *Saccharomycetes*, and *Calosphaeriales*), 7 for the C stage (e.g., *Stachybotrys*, *Coniolariella*, and *Xylariaceae*), 2 for the D stage (e.g., *Didymellaceae* and *Dothideomycetes*), and 12 for the E stage (e.g., *Alternaria*, *Filobasidium*, and *Filobasidiaceae*; Figure 3D). For bacteria, LEfSe showed that 67 taxa had significant differences. Among them, there were 27 taxa for the A stage (e.g., *Bacteroides*, *Bacilli*, and *Lachnospiraceae*), 16 for the B stage (e.g., *Sphingobacterium*, *Pseudomonas*, and *Gammaproteobacteria*), 1 for the C stage (e.g., *Brevundimonas diminuta*), 9 for the D stage (e.g., *Rhodopseudomonas*, *Sphingomonas*, and *Massilia*), and 14 for the E stage (e.g., *Microbacteriaceae*, *Actinobacteria*, and *Brevundimonas*; Figure 3E). These results indicated that there were significant differences in grape epidermis microbes at different developmental stages.

### Dynamics of Core Microbiome During Grape Development

Here, we prioritized the core microbiome to further investigate the fungal and bacterial community succession of grape epidermis. At the genus level, the most important taxa were first identified according to the abundance-occupancy distribution, as the core microorganisms of the grape epidermis. The relative abundance of the fungal core taxa reached 58.58 ± 2.39%, including some filamentous fungi (*Aspergillus*, *Rhizopus*, *Alternaria*, and *Penicillium*) and yeasts (*Clavispora*, *Candida*, and *Cyberlindnera*; Supplementary Table 2). The relative abundance of the bacterial core taxa reached 71.73 ± 3.08%, including the common lactic acid bacteria (*Leuconostoc* and *Lactobacillus*) and spoilage bacteria (*Gluconobacter*) in wine (Supplementary Table 2).

**FIGURE 4:**
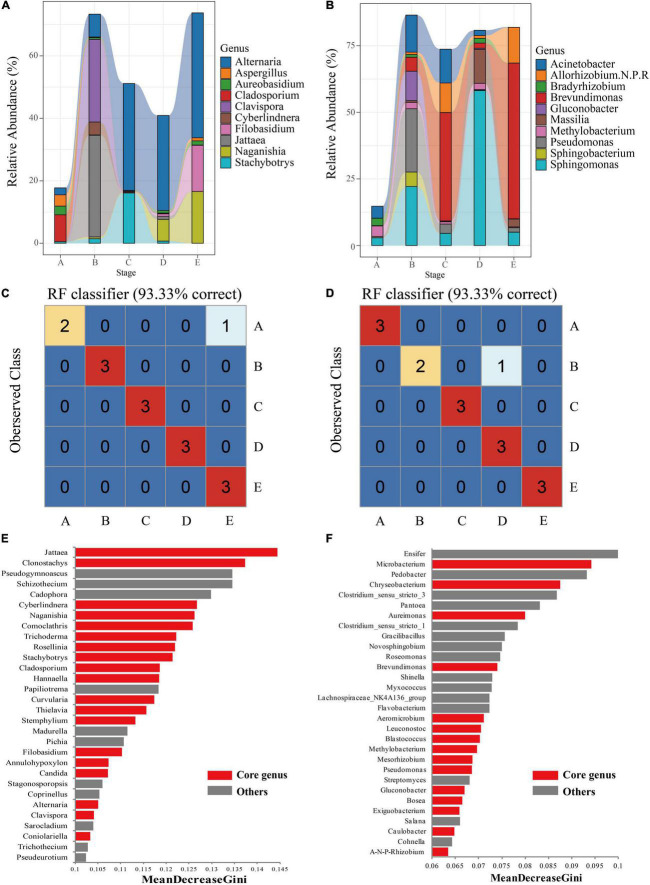
The core microbiome of grape epidermis at different developmental stages. The relative abundance of the core taxa of fungi **(A)** and bacteria **(B)** changed with development (relative abundance top 10 shown). The results of the Random Forest model showed that the microbiota of fungi **(C)** and bacteria **(D)** could distinguish the developmental stages of grapes. Important taxa of fungi **(E)** and bacteria **(F)** are shown based on the MeanDecreaseGini value. A, fruit setting; B, early veraison; C, end veraison; D, mid maturity; E, harvest.

Tracing the core taxa revealed significant temporal dynamics and succession associated with berry development. Overall, veraison appeared to be a key stage where the core taxa were different from other stages. For example, the fungal core taxa, *Clavispora* and *Jattaea* only existed with higher abundance at the B stage, while *Alternaria*, *Filobasidium*, and *Naganishia* gradually accumulated abundance from the C stage (Figure 4A). It was noteworthy that a few fungal core taxa only appeared at specific developmental stages. *Cyberlindnera* and *Comoclathris* could be detected from the B stage. *Rosellinia*, *Jattaea*, and *Coniolariella* were detected only in the middle stage of berry development (Supplementary Table 2). For bacterial core taxa, the dominant genus *Brevundimonas* began to accumulate with higher abundance from the B stage. *Acinetobacter* existed with high abundance before the E stage, especially veraison (Figure 4B). Similarly, a few core taxa appeared only at specific development stages. *Azorhizobium* could only be detected from the B stage. *Caedibacter* only existed with low abundance in the middle stage of berry development (Supplementary Table 2).

To verify the robustness of these observations in the berry growth cycle, random forest supervised learning models were used to classify the samples and determine which taxa explain the strongest variations in the grape development stage. Fungi and bacterial communities had a high discriminative power, with a resolution of 93.33% for different stages of the samples (Figures 4B,C). Among them, fungi and bacteria made misjudgments (class error = 33.33%) on the samples of the A stage and the B stage, respectively. The important features identified at different development stages of berries included not only core taxa but also non-core taxa. For fungal communities, filamentous fungi (e.g., *Jattaea*, *Clonostachys*, and *Trichoderma*) and fermenting yeasts (e.g., *Cyberlindnera*) within the core were top features in the classification models (Figure 4E). Some non-core taxa were also important for the models, such as *Pseudogymnoascus* and *Pichia*, which had relatively high abundance at the B stage compared with other stages, which was the key taxa distinguishing the B stage (Supplementary Table 3). *Madurella* could only be detected at the early stages of berry development, which was also an important feature of the classification model (Supplementary Table 3). For bacterial communities, *Microbacterium*, *Chryseobacterium*, and *Aureimonas* of the core taxa were top features (Figure 4F). Compared with other stages, non-core taxa *Ensifer* had the highest abundance at the C stage, which was the key taxa distinguishing the C stage; *Pedobacter* also had the highest abundance at the E stage (Supplementary Table 3). When the core taxa of fungi and bacteria were used to build a random forest classification model, all samples from different stages could be correctly identified (Supplementary Figure 2), indicating that the developmental stages of grapes could be distinguished based on the core taxa.

### Co-occurrence Analysis of the Relationship Between Microorganisms During Grape Development

**FIGURE 5:**
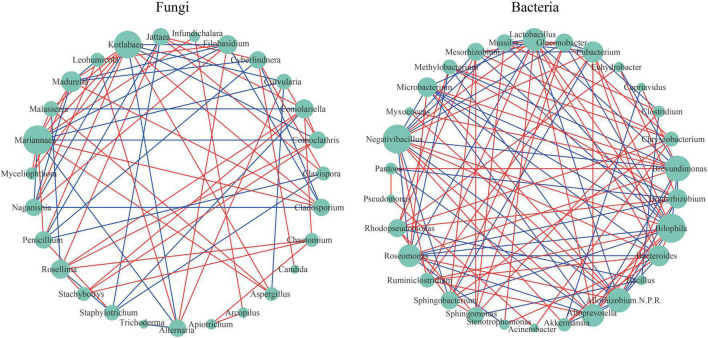
Network visualization shows co-occurrence and exclusion correlations among fungi and among bacteria at the genus level. The green circular nodes represent microbial taxa. Direct connections between nodes indicated strong correlations (Spearman correlation coefficient, *r* ≥ 0.6; *p* < 0.05). The color of the edges describes the positive correlation (red) or the negative correlation (blue). The size of the node is directly proportional to the degree of interconnection.

Microbial co-occurrence network analysis is a common tool to study the microbial community structure and the inside interactions. Figure 5 shows different topological structures of fungi and bacteria. The network of bacteria (network density = 0.253) was denser than that of fungi (network density = 0.202). For fungi taxa, *Mariannaea* had the highest connectivity (degree = 13). For example, *Mariannaea* was positively correlated with *Malassezia*, *Madurella*, *Kotlabaea*, *Aspergillus*, *Cladosporium*, *Penicillium*, *Myceliophthora*, and *Infundichalara*, and negatively correlated with *Alternaria*, *Comoclathris*, *Curvularia*, *Naganishia*, and *Filobasidium* (Figure 5). *Trichoderma* and *Candida* had the lowest connectivity (degree = 1). For example, *Trichoderma* was only negatively correlated with *Alternaria*, and *Candida* was only positively correlated with *Cyberlindnera* (Figure 5). For bacterial taxa, *Bilophila* and *Negativibacillus* had the highest connectivity (degree = 15), and there was a positive correlation between them. For example, *Bilophila* was positively correlated with *Ruminiclostridium*, *Lactobacillus*, *Eubacterium*, *Bacteroides*, *Alloprevotella*, and *Akkermansia*, and negatively correlated with *Sphingobacterium*, *Roseomonas*, *Microbacterium*, *Mesorhizobium*, *Massilia*, *Gluconobacter*, *Allorhizobium-Neorhizobium-Pararhizobium-Rhizobium*, and *Brevundimonas*; *Negativibacillus* was positively correlated with *Ruminiclostridium*, *Eubacterium*, *Bacteroides*, *Alloprevotella*, *Akkermansia*, and *Lactobacillus*, and negatively correlated with *Sphingobacterium*, *Microbacterium*, *Mesorhizobium*, *Massilia*, *Gluconobacter*, *Brevundimonas*, *Allorhizobium-Neorhizobium-Pararhizobium-Rhizobium*, and *Roseomonas* (Figure 5). *Pseudomonas*, *Acinetobacter*, *Myxococcus*, and *Cupriavidus* had the lowest connectivity (degree = 2). For example, *Pseudomonas* was positively correlated with *Sphingobacterium* and *Sphingobacterium*; *Acinetobacter* was positively correlated with *Pseudomonas* and *Chryseobacterium*; *Myxococcus* was positively correlated with *Brevundimonas* and *Allorhizobium-Neorhizobium-Pararhizobium-Rhizobium*; *Cupriavidus* was positively correlated with *Brevundimonas* and negatively correlated with *Akkermansia*.

### Effect of Vineyard Weather on Microbial Community of Grape Epidermis

**FIGURE 6:**
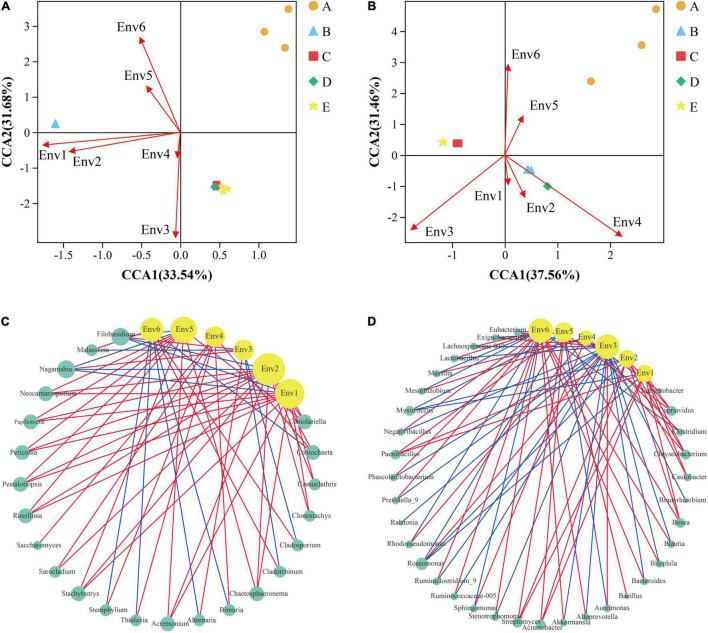
Statistically correlation among grape microorganisms and vineyard weather was observed during grape development. CCA reflects the relationship between meteorological parameters and the diversity of fungi **(A)** and bacteria **(B)** during grape development. Network analysis reflected the co-occurrence relationship between the fungi **(C)** and bacteria **(D)** taxa and meteorological parameters. The yellow circular nodes represent meteorological parameters. The green circular nodes represent microbial taxa. Direct connections between nodes indicated strong correlations (Spearman correlation coefficient, *r* ≥ 0.5; *p* < 0.05). The color of the edges represents positive correlation (red) or negative correlation (blue). The size of the node is directly proportional to the degree of interconnection. Env1, average high temperature; Env2, average low temperature; Env3, relative humidity; Env4, precipitation; Env5, average ground temperature; Env6, sunshine time. A, fruit setting; B, early veraison; C, end veraison; D, mid maturity; E, harvest.

The microbial community of the grape epidermis was closely associated with vineyard weather. During the grape growth cycle, all weather parameters displayed significant differences between the phenological stages (Supplementary Table 4). The phase-dependent pattern of the grape epidermal microbial community also indicated that longitudinal environmental conditions were likely responsible for structuring the microbial communities. CCA can be used to explore the influence of vineyard weather on the variation of grape epidermal microbial communities. Figures 6A,B show that vineyard weather had less effect on fungal and bacterial diversity of the A stage. The fungal diversity of the B stage was mainly affected by average high temperature and average low temperature; while the maturation process (C–E stage) was mainly affected by relative humidity and precipitation (Figure 6A). The bacterial diversity of the B and D stages was mainly affected by precipitation; the C and E stages were mainly affected by relative humidity (Figure 6B).

The microbial community of grape epidermis was closely associated with each meteorological parameter. Network analysis was conducted to explore the co-occurrence patterns between grape epidermis microorganisms and meteorological parameters, based on the strong correlation coefficients (Spearman correlation coefficient, *r* ≥ 0.5; *p* < 0.05). The connected module between bacteria (average degree = 12.2, Figure 6D) and meteorological parameters was observed more closely than fungi (average degree = 9.8, Figure 6C). For the fungal taxa, the average high temperature, the average low temperature, and the average ground temperature had a higher connection density with the fungi taxa (degree > 10). For example, the average high temperature was positively correlated with *Stachybotrys* and negatively correlated with *Naganishia*; the average low temperature was positively correlated with *Coniolariella* and negatively correlated with *Flobasidium*; the average ground temperature was positively correlated with *Rosellinia* and negatively correlated with *Naganishia* (Figure 6C). For bacterial taxa, the relative humidity, the average ground temperature, and sunshine hours had a higher connection density with the bacterial taxa (degree > 10). For example, relative humidity was positively correlated with *Aureimonas* and negatively correlated with *Lactobacillus*; average ground temperature was positively correlated with *Acinetobacter* and negatively correlated with *Roseomonas*; sunshine hours were positively correlated with *Stenotrophomonas* and negatively correlated with *Sphingomonas* (Figure 6D).

## Discussion

### Microbial Diversity in Grape Epidermis

The grape surface is conducive to the growth of different types of microorganisms, so it is very important to understand the diversity and community functions of grape microflora. In this study, a total of 5 fungal phyla were detected, of which Ascomycota (88.89%) and Basidiomycota (9.71%) were the main ones, followed by Mortierellomycota (0.52%), Mucoromycota (0.47%), and Rozellomycota (<0.01%). It has been reported in the literature that Ascomycota and Basidiomycota were the dominant groups of grape epidermal microorganisms. Gao et al. (2019) studied the microbial diversity of wine grape epidermis in different regions of Xinjiang, and detected 3 fungal phyla, mainly Ascomycota and Basidiomycota, followed by Zygomycota. Compared to those at the phylum level, our study detected more fungal groups. Fungi were the main causes of common grape diseases, such as Phylloxera (*Daktulosphaera vitifoliae*), Down mildews (*Plasmopara viticola*), Powder mildews (*Erysiphe Necator*), Grey mold (*Botryotinia fuckelina*), and Anthracnose (*Colletotrichum gloeosporides*). In this study, *Colletotrichum gloeosporoides* and *Erysiphe Necator* (relative abundance < 0.01%) were detected at the B and E stages, respectively, but no disease was observed in vineyards. In addition, many types of yeasts were detected, such as *Hanseniapora*, *Candida*, *Pichia*, *Saccharomyces*, *Rhodotorula*, and *Wickerhamomyces*. For bacteria, a total of 27 phyla were detected, mainly Proteobacteria (82.31%), Firmicutes (7.38%), Bacteroidetes (4.56%), Actinobacteria (2.99%), and Cyanobacteria (1.40%). Portillo et al. (2016) studied the bacterial diversity of Grenache and Carignan epidermal bacteria in Priorat area; only 14 phyla were detected, but the main bacteria were Proteobacteria, Firmicutes, Actinobacteria, and Bacteroidetes. Bacteria are also the main cause of common grape diseases, such as Pierce’s Disease (*Xylella fastidiosa*). The bacterial pathogens in grapes were not detected in this study. In addition, many types of lactic acid bacteria, such as *Lactobacillus*, *Leuconostoc*, *Pediococcus*, and *Weissella*, were detected. Although our data revealed the complex and abundant grape microbial structure, there are still hidden microbial species that cannot be exposed, as confirmed by the rarefaction curve (Figure 1).

Biological control is becoming the main method of current plant disease management. In this study, the most potential fungal antagonist *Trichoderma* was detected, and *Pichia*, *Aspergillus*, and *Penicillium* are being studied. *Alternaria*, as the dominant genus in this study, was also found among the dominant members of the grapevine community in different regions and varieties (Gavin et al., 2017; Knapp et al., 2021). Many of its species are considered to be cosmopolitan saprobes, endophytes, pathogens, or the causal agents of postharvest rots of numerous agronomic plants (Armitage et al., 2015). In the analysis of the interaction between microorganisms, it was found that *Trichoderma* and *Alternaria* were negatively correlated during grape development (Figure 5). This phenomenon was supported by the test results of Bhattacharya and Chakraborty (2020). In addition, bacteria also have a biological control effect. According to previous studies, the most well-known and reported bacterial antagonists are species of *Pseudomonas*, *Burkholderia*, *Bacillus*, *Serratia*, and *Pantoea* (Trotel-Aziz et al., 2008; Bulgari et al., 2011). For example, Sawant et al. (2016) isolated multiple *Bacillus* species from vineyards that effectively inhibited the enrichment of *Colletortrichum gloeosporiodes*, *Erysiphe necator*, and *Plasmopara viticola*. This also explained why anthracnose and powdery mildew disease phenomena were not observed in this study. Our data contribute to the characterization of the grape biodiversity and to the analysis of biomarkers with the potential to unveil the vine health status. This will have a significant influence on the vine performance and also on the wine quality.

### Grape Epidermal Microbes in Response to the Development Stages of Berries

Microbiome succession is associated with plant development. Studies have shown that phyllosphere and rhizospheric fungal and bacterial communities of a wide range of plants, such as lettuce, alfalfa, switchgrass, and miscanthus, change according to plant development stages (Mougel et al., 2006; Rastogi et al., 2012; Grady et al., 2019). The nutrient composition of grapes is one of the important factors determining microorganisms on the grape surface (Ranade et al., 2021). In the early stages of development, the metabolism of berries is active, the acid content increases rapidly, and a small amount of sugar (not more than 10–20 g/L) appears. The acidic conditions in the early stage of grape growth are favorable for the colonization of most bacteria and a few fungi (Setati et al., 2012). In this study, the bacterial diversity of the A stage was significantly higher than that of fungi (Figure 3A). Veraison marks the beginning of grape ripening, which is a critical time point for grape metabolism and growth. Besides color changes caused by the production and accumulation of anthocyanins, the berry softens with the degradation of pectin and cellulose, the decrease of acidity, and the accumulation of sugar (Fasoli et al., 2016). All these factors create a more favorable environment for the colonization of grape epidermal microorganisms. In this study, veraison was the key stage of the change of epidermal microbial diversity. Compared with the A stage, the total relative abundance of fungal and bacterial core communities at the B stage was significantly higher. For example, the total relative abundance of the fungal core community was 30.76 ± 5.54 and 79.74 ± 2.63% at the A and B stages, respectively, and the bacterial core community was 19.11 ± 3.93 and 91.50 ± 1.08%, respectively (Supplementary Table 2). Similarly, the core members had undergone significant changes in Veraison. For example, the relative abundance of *Alternaria* at the A and C stages was 2.21 and 34.24%, and the relative abundance of *Brevundimonas* at the A and C stages was 0.05 and 40.64% (Supplementary Table 2). In addition, some taxa existed only at specific stages, such as *Mariannaea* and *Negativibacillus* only at the A stage. Studies have shown that plants can recruit microbes to participate in the key physiological processes and drive microbial assemblages to respond to biotic or abiotic stresses and improve environmental fitness (Hu et al., 2018). Our data may indicate how plant-driven microbes respond to environmental changes.

### Grape Epidermal Microbes in Response to Vineyard Weather

Alongside the influences of changing metabolism and physiology during plant development, the grape epidermal microbiome is also subject to dynamic ecological conditions (Liu et al., 2019; Ranade et al., 2021). The microbial biogeography of grape berries is affected by climate, and populations are associated with specific climatic conditions, indicating a link between vineyard environmental conditions and microbial communities (Bokulich et al., 2014). In this study, water status (relative humidity and precipitation) had the greatest impact on fungal and bacterial diversity throughout the berry phenology period (Figure 6). For example, higher solar radiation and temperature, decreased precipitation, increased evaporation and transpiration, and lower relative soil moisture were observed from the A stage to the B stage in the vineyard, and this water stress event coincided with dramatic changes in the microbiome during the period discussed above (Figure 3A). Water status in the vineyard can condition the microbial populations of grape and must be at regional scales. Studies have shown that precipitation and humidity correlate with the presence of filamentous fungi (e.g., *Penicillium*) and yeasts (e.g., *Saccharomyces*, *Hanseniaspora*, and *Metschnikowia*; Bokulich et al., 2014; Jara et al., 2016). In this study, relative humidity was positively correlated with *Alternaria* and *Aureimonas*, and negatively correlated with *Malassezia* and *Lactobacillus*; precipitation was positively correlated with *Saccharomyces* and *Sphingomonas* (Figures 6C,D). In addition, water stress can trigger a large number of physiological reactions in grapevines, and disturb the water supply relationship of grapevines, the accumulation of grape chemical components, and the synthesis of secondary metabolites (Anjum et al., 2011; Hochberg et al., 2013), which may also affect the colonization of grape epidermis-related species, but further research is needed.

In summary, the microbial diversity of the Cabernet Sauvignon epidermis in the Wuhai region of China showed a downward trend during the development of berries. This diversity was mainly associated with berry development and the weather in the vineyard. Grapes recruited microbes according to their developmental stages, but retained the core species. In conclusion, this study provides in-depth information regarding the differences in the microbial communities on the surface of grapes during the developmental stages. Future research should further investigate the changes and functions of microbial communities in other habitats of grapevines (root zone soil, root system, leaves, and flowers) in order to increase our understanding of vineyard microbial ecology. This study will help improve vineyard management techniques, such as the use of biological control strategies to reduce pesticide costs, in order to maintain and produce healthy, high-quality grapes, allowing the expression of the regional character of the wine.

## Data Availability Statement

The original contributions presented in this study are included in the article/Supplementary Material, further inquiries can be directed to the corresponding authors. The data presented in the study are deposited in the NCBI repository, accession number: PRJNA766154.

## Author Contributions

RW and NC collected wine grape samples, compiled the figures and table, and wrote the manuscript. YD and LW conceived the framework of the manuscript. YL, FG, and LZ conducted the bioinformatic analysis of data and provided advice and constructive critiques. HL and HW supervised the research activities. All authors reviewed the manuscript.

## Conflict of Interest

The authors declare that the research was conducted in the absence of any commercial or financial relationships that could be construed as a potential conflict of interest.

## Publisher’s Note

All claims expressed in this article are solely those of the authors and do not necessarily represent those of their affiliated organizations, or those of the publisher, the editors and the reviewers. Any product that may be evaluated in this article, or claim that may be made by its manufacturer, is not guaranteed or endorsed by the publisher.
