# Supplementary material for: Diversity and Dynamics of Epidermal Microbes During Grape Development of Cabernet Sauvignon (Vitis vinifera L.) in the Ecological Viticulture Model in Wuhai, China
Source: Front Microbiol. 2022 Jun 30;13:935647. doi: 10.3389/fmicb.2022.935647 (PMC9280189; doi:10.3389/fmicb.2022.935647)
Supplement: Supplementary file 1 [file Data_Sheet_1.docx]

Supplementary Figure


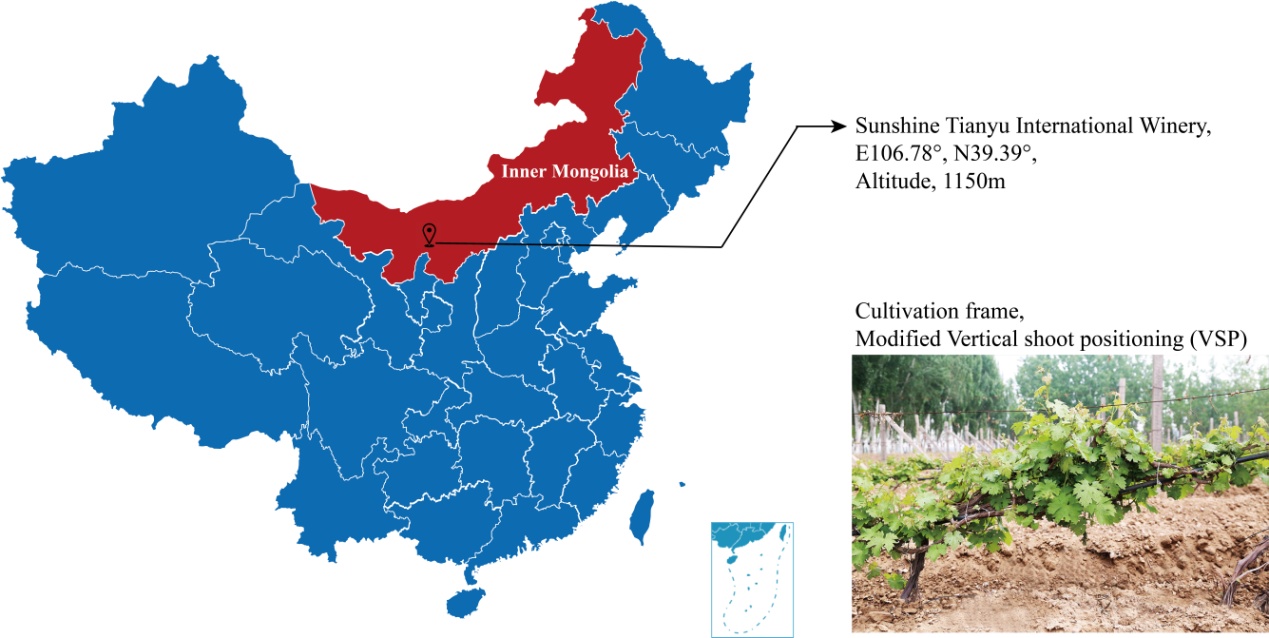


Figure S1. Geographical location information of Sunshine Tianyu International Winery vineyards. Sunshine Tianyu International Winery is located in Wuhai City, Inner Mongolia, on the upper reaches of the Yellow River, surrounded by the Ulan Buh Desert, Hobq Desert and Mu Us Desert. Wuhai City belongs to temperate continental climate, with dry in spring, high temperature and less rain in summer, large temperature difference between day and night, long sunshine time, rich visible light resources, and annual average precipitation of 200 mm.


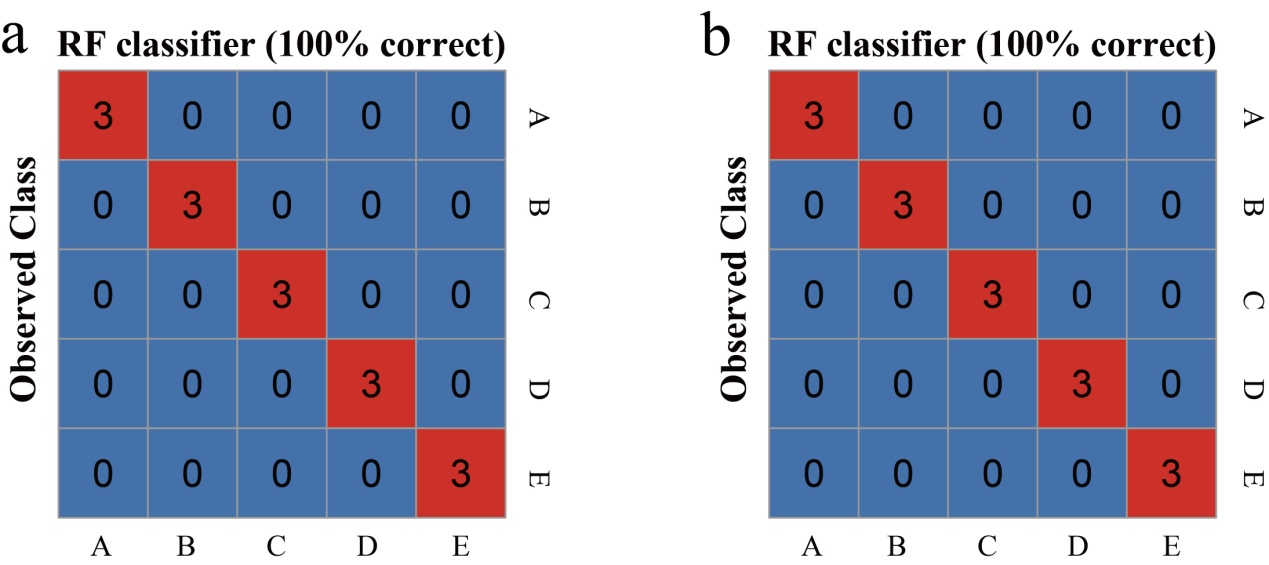


Figure S2. The core taxa of fungal (a) and bacteria (b) were used to build random forest models, all of which correctly identified samples from different stages. A-E represent stages of grape berry development. A, fruit setting; B, Early veraison; C, end veraison; D, mid maturity; E, Harvest.
